# Increasing brain glucose metabolism by ligustrazine piperazine ameliorates cognitive deficits through PPARγ-dependent enhancement of mitophagy in APP/PS1 mice

**DOI:** 10.1186/s13195-022-01092-7

**Published:** 2022-10-11

**Authors:** Zongyang Li, Xiangbao Meng, Guoxu Ma, Wenlan Liu, Weiping Li, Qian Cai, Sicen Wang, Guodong Huang, Yuan Zhang

**Affiliations:** 1 Department of Neurosurgery, Shenzhen Key Laboratory of Neurosurgery, Shenzhen Institute of Translational Medicine, the First Affiliated Hospital of Shenzhen University, Shenzhen Second People’s Hospital, No. 3002 Sungang Westroad, Futian District, Shenzhen, 518035 China; 2 College of Pharmacy, Jinan University, No. 855 Xingye Avenue East, Panyu District, Guangzhou, 511486 China; 3 Institute of Medicinal Plant Development, Chinese Academy of Medical Sciences and Peking Union Medical College, No. 151, Malianwa North Road, Haidian District, Beijing, 100193 China; 4 School of Medicine, Xi’an Jiaotong University, No.76, Yanta Westroad, Xi’an, 710061 China

**Keywords:** PPARγ, PINK1/Parkin, Mitophagy, Mitochondria, Alzheimer’s disease

## Abstract

PPARγ agonists have been proven to be neuroprotective in vitro and in vivo models of Alzheimer’s disease (AD). In the present study, we identified ligustrazine piperazine derivative (LPD) as a novel PPARγ agonist, which was detected by a dual-luciferase reporter assay system. LPD treatment dose-dependently reduced Aβ40 and Aβ42 levels in PC12 cells stably transfected with APP695swe and PSEN1dE9. Intragastric administration of LPD for 3 months dose-dependently reversed cognitive deficits in APP/PS1 mice. LPD treatment substantially decreased hippocampal Aβ plaques in APP/PS1 mice and decreased the levels of Aβ40 and Aβ42 in vivo and in vitro. Moreover, LPD treatment induced mitophagy in vivo and in vitro and increased brain ^18^F-FDG uptake in APP/PS1 mice. LPD treatment significantly increased OCR, ATP production, maximal respiration, spare respiratory capacity, and basal respiration in APP/PS1 cells. Mechanistically, LPD treatment upregulated PPARγ, PINK1, and the phosphorylation of Parkin (Ser65) and increased the LC3-II/LC3-I ratio but decreased SQSTM1/p62 in vivo and in vitro. Importantly, all these protective effects mediated by LPD were abolished by cotreatment with the selective PPARγ antagonist GW9662. In summary, LPD could increase brain glucose metabolism and ameliorate cognitive deficits through PPARγ-dependent enhancement of mitophagy in APP/PS1 mice.

## Introduction

Alzheimer’s disease (AD) is a severe neurodegenerative disease characterized by an accumulation of senile plaques composed of amyloid-β (Aβ) peptide and neurofibrillary tangles, which are comprised of hyperphosphorylated tau protein in the brain [9]. However, the pathogenesis of AD is not fully understood. Epidemiological studies support a connection between type 2 diabetes mellitus (T2DM) and Alzheimer’s disease [2, 3, 28]. Central glucose dysregulation is a fundamental pathological hallmark of AD [7, 15].

Typically, glucose enters the brain through glucose transporters and is metabolized to ATP via the tricarboxylic acid cycle and the electron transport chain within mitochondria [19]. Functional glucose transporters and mitochondria are two key elements of cerebral energy homeostasis [14]. These elements are of utmost importance, as both glucose transportation abnormalities and mitochondrial dysfunction have a pathological role in AD [1]. Mitochondrial homeostasis is temporally and spatially regulated by mitophagy [6]. Mechanistically, the ubiquitin kinase PINK1 localizes to dysfunctional mitochondria, where it recruits and activates Parkin by phosphorylation on Ser65, leading to lysosomal engulfment and elimination of dysfunctional mitochondria [17, 29]. PINK1 and Parkin deficiency results in the accumulation of dysfunctional mitochondria in the neurons of patients with AD and in rodent models [8, 12]. Therefore, strategies to counteract glucose dysmetabolism encompassing diminished glucose transporters and/or defective mitophagy are warranted.

Peroxisome proliferator-activated receptor gamma (PPARγ) belongs to the nuclear hormone receptor superfamily. It plays central roles in glucose metabolism [27]. Pan-PPAR modulation could effectively protect APP/PS1 mice from amyloid deposition and cognitive deficits [16]. PPARγ agonists, including rosiglitazone and pioglitazone, have shown beneficial effects on cognitive deficits in transgenic mouse models of AD [11, 37]. Interestingly, we have recently reported that 15-deoxy-Δ12,14-prostaglandin J2, an endogenous PPARγ agonist, could ameliorate cognitive deficits seen in APP/PS1 mice and decreased extracellular Aβ plaques in the hippocampus [21]. Hence, targeting PPARγ may represent a potential therapeutic strategy for the treatment of AD [10].

Ligustrazine is an alkaloid extracted from the herbal medicine *Ligusticum chuanxiong hort*, which has been widely used to treat cerebrovascular diseases in Asia for centuries [32]. Ligustrazine has been shown to activate PPARγ and promote mitophagy by inducing Parkin translocation to the mitochondria [38, 40]. Importantly, ligustrazine improves cognitive impairment in rodent models of AD [13, 35], suggesting that ligustrazine may become a novel drug candidate for the treatment of AD. However, the short elimination half-life of ligustrazine seriously limits its application in clinical practice [41].

In this study, we synthesized a ligustrazine piperazine derivative (LPD). However, whether LPD has protective effects on AD remains uncertain. Here, we provide evidence that LPD is a novel PPARγ agonist and ameliorates cognitive deficits through PPARγ-dependent enhancement of mitophagy and glucose metabolism in the hippocampus of APP/PS1 mice.

## Materials and methods

### Materials

Rosiglitazone (HY-17386, purity = 99.90%), ligustrazine (HY-N0264, purity = 99.91%), GW9662 (HY-16578, purity = 99.83%), and Mdivi-1 (HY-16578, purity = 99.73%) were supplied by MedChem Express (Shanghai, China). Bovine serum albumin (BSA), Triton X-100, isoflurane, and paraformaldehyde were purchased from Sigma-Aldrich (MO, USA). Dulbecco’s modified Eagle’s medium (DMEM), fetal bovine serum (FBS), penicillin, streptomycin, Lipofectamine 6000, RIPA buffer, phosphate-buffered saline (PBS), saline, and phospho-Parkin (Ser65) polyclonal antibody (PA5-114616), as well as human enzyme-linked immunosorbent assay (ELISA) kits for Aβ40 (KHB3481) and Aβ42 (KHB3544), were all obtained from Invitrogen (CA, USA). Anti-Aβ (ab201060), anti-PPARγ (ab178860), anti-PINK1 (ab23707), anti-LC3B (ab192890), anti-SQSTM1/p62 (ab109012), and anti-beta tubulin (ab6046) antibodies, as well as Alexa Fluor® 647-conjugated goat anti-rabbit IgG H&L (ab150083), HRP-conjugated goat anti-rabbit IgG H&L (ab6721), and HRP-conjugated goat anti-mouse IgG H&L (ab6789), were all purchased from Abcam (CA, USA). The bicinchoninic acid (BCA) kit and enhanced chemiluminescence (ECL) kit were purchased from Pierce Biotechnology (IL, USA). The 18-Fluoro-6-deoxyglucose (^18^F-FDG) was obtained from Union Hospital Affiliated to Tongji Medical College, Huazhong University of Science and Technology.

### Synthesis of ligustrazine piperazine derivative

N-Monosubstituted piperazine (10 mmol) and 2-chloromethyl-3,5,6-trimethylpyrazine hydrochloride (10 mmol) were added to 70 mL of toluene, followed by addition of 40 mmol of NaHCO_3_ and a catalytic amount of NaI. The mixture was heated and refluxed for 10 h. TLC showed that the reaction was complete. After filtering, the filter cake was washed 3 times with a small amount of toluene, combined with filtrate, vacuum distilled to obtain oil, fast column separated to produce a light yellow powder, and recrystallized with n-hexane to obtain white crystal 1-benzhydryl-3-((3,5-dimethylpyrazin-2-yl)methyl)hexahydropyrimidine (LPD).

### Cell culture

PC12 cells were obtained from the Cell Resource Center of the Institute of Basic Medical Sciences, Peking Union Medical College and Chinese Academy of Medical Sciences (Beijing, China). PC12 cells were maintained in DMEM supplemented with 10% FBS, 100 U/mL penicillin, and 100 μg/mL streptomycin at 37 °C and 5% CO_2_ in a humidified incubator (Thermo Scientific, Langenselbold, Germany).

### Dual-luciferase reporter assay

To determine whether LPD is a novel PPARγ agonist, the PPRE-TK-luc vector (1 μg) and PPARγ expression plasmid (1 μg) were cotransfected with 20 ng of pRL-TK (Promega, WI, USA) into PC12 cells using Lipofectamine 6000 when the cells reached 80% confluence. To construct the PPARγ expression plasmid, human PPARγ (NCBI reference sequence: NM_001354666) was PCR amplified and then fused with the GV230 vector (Shanghai Genechem Technology Co., Ltd., Shanghai, China). To construct the PPRE luciferase reporter plasmid, human PERM1 (NCBI reference sequence: NM_001291366.2) was PCR amplified and subcloned into the GV238 luciferase reporter vector (Shanghai Genechem Technology Co., Ltd., Shanghai, China). pRL-TK was used to adjust for transfection efficiency. After 48 h of transfection, PC12 cells were treated with various concentrations of LPD (2.5, 5, 10, and 20 μM) or ligustrazine (2.5, 5, 10, and 20 μM) or rosiglitazone (40 μM) or cotreated with LPD (20 μM) and GW9662 (10 μM) for 12 h. Luciferase activity was measured using a dual-luciferase reporter assay system (Promega, WI, USA) with a Glomax 20/20 luminometer (Turner Designs, CA, USA). The luciferase activity was normalized to Renilla luciferase activity. Cell viability was determined using a Cell Counting Kit-8 (Dojindo Laboratories, Kumamoto, Japan), according to the manufacturer’s instructions.

### APP695swe/PSEN1dE9-overexpressing stable cell line

We previously established stable APP695swe-transfected PC12 cells [26]. To generate APP/PS1 double-overexpressing cells, human PSEN1 cDNA (NCBI reference sequence: NM_000021.4) was amplified through PCR. Mutant human PSEN1 (PSEN1dE9) was constructed and subcloned into the GV208 vector (Shanghai Genechem Technology Co., Ltd., Shanghai, China). The PSEN1dE9 plasmid was cotransfected with the framework plasmid vector pHelper 1.0 and pHelper 2.0 into HEK293T cells to produce Lenti-PSEN1dE9 (Shanghai Genechem Technology Co., Ltd., Shanghai, China). APP695swe stably transfected PC12 cells were seeded on six-well plates and infected with Lenti-PSEN1dE9 when the cells reached 80% confluence. Puromycin (2 μg/mL) was added, and drug-resistant cells were collected after 2 weeks for single-cell cloning. Steadily transfected cells were maintained in puromycin at a final concentration of 1 μg/mL. Resistant clones were analyzed by laser confocal microscopy and western blotting to confirm the overexpression of APP and PSEN1. The APP/PS1 cells (1.5 × 10^4^ per well) were plated in 6-well plates and cultured for another 24 h. The APP/PS1cells were incubated with ligustrazine (20 μM) or GW9662 (10 μM) or different concentrations of LPD (2.5, 5, 10, and 20 μM) or cotreated with LPD (20 μM) and GW9662 (10 μM) for 24 h. The levels of Aβ40 and Aβ42 were detected using the respective ELISA kits. Cell viability was determined using the Cell Counting Kit-8, according to the manufacturer’s instructions.

### Seahorse assay

Mitochondrial oxidative phosphorylation capacity was determined as the uncoupled oxygen consumption rate (OCR) using a Seahorse XF96 extracellular flux analyzer (Seahorse Biosciences, MA, USA). Briefly, the APP/PS1 cells (5 × 10^4^ per well) were plated in XF96 extracellular flux assay plates and cultured for another 24 h. The APP/PS1 cells were incubated with LPD (20 μM) or cotreated with LPD (20 μM) and GW9662 (10 μM) or cotreated with LPD (20 μM) and Mdivi-1 (20 μM). After 24 h, the medium was replaced with XF Assay Medium (Seahorse Bioscience MA, USA). After the cells were incubated in a CO_2_-free incubator at 37 °C for 30 min, basal levels were measured with no additives. For OCR detection, oligomycin, FCCP, and rotenone/antimycin A were added at final concentrations of 1 μM, 0.3 μM, and 0.1 μM, respectively. Three separate measurements were taken after each of the above reagents was added. Triplicate experimental wells were examined, and the results were plotted using Seahorse software.

### Animals and ethical considerations

All animal protocols were approved by the ethics committee of Shenzhen Second People’s Hospital. The experiments were conducted in compliance with the Guide for the Care and Use of Laboratory Animals. All efforts were made to reduce the number of animals used and minimize animal suffering in the experiments. Six-month-old male Swedish mutant APP (APP695swe)/PS1 (PSEN1dE9) transgenic mice and age-matched male C57BL/6N wild-type (WT) mice were obtained from Beijing HFK Bioscience Co., Ltd. (Beijing, China). The animals were housed in a specific pathogen-free animal facility at a constant room temperature of 22 ± 1 °C and 50 ± 10% relative humidity with 12-h light/12-h dark cycles. Access to standard rodent chow and water was available ad libitum.

### Animal treatments

After adaptation for 7 days, 60 APP/PS1 mice were randomly assigned to 6 groups for dose selection: vehicle, rosiglitazone (10 mg/kg/day), GW9662 (5 mg/kg/day), LPD (5 mg/kg/day), LPD (10 mg/kg/day), and LPD (20 mg/kg/day). Twenty age-matched C57BL/6N WT mice were randomly assigned to vehicle and LPD (20 mg/kg/day) groups. Each group consisted of 10 mice. LPD, rosiglitazone, and GW9662 were prepared in 5% DMSO and 95% saline containing 20% SBE-β-CD and stored at 4 °C until use. The mice were intragastrically administered LPD, rosiglitazone, GW9662, or an equivalent volume of vehicle for 3 months.

In the following experiments, 60 APP/PS1 mice were randomly assigned into 3 groups: vehicle, LPD (10 mg/kg/day), and GW9662 (5 mg/kg/day) + LPD (10 mg/kg/day). Each group consisted of 20 mice. Twenty age-matched C57BL/6N WT mice were used as controls. Each mouse was intragastrically administered either vehicle, LPD, or GW9662 every day from the age of 6 months for a total period of 3 months.

The body weights of the mice were monitored weekly. The volumes of LPD, GW9662, and vehicle were adjusted according to the body weights of the mice. After completion of drug treatments, 10 mice were randomly selected from each group for the Morris water maze test, and the other mice (*n* = 10 per group) were subjected to micropositron emission tomography (microPET). All mice were sacrificed with an overdose of isoflurane anesthetic, and 14 mice were randomly selected from each group and perfused transcardially with saline using a syringe infusion pump at a 5-min/min rate for 5 min. The brain tissue was quickly removed following decapitation, and the hippocampus was then dissected and frozen for ELISA (*n* = 8 per group) and western blotting (*n* = 6 per group). The other mice (*n* = 6 per group) were perfused with 4% paraformaldehyde following saline perfusion, and brain tissue (*n* = 3 per group) was collected for immunofluorescence. The hippocampi (*n* = 3 per group) were then dissected and further processed with transmission electron analysis.

### Morris water maze

The Morris water maze test was performed to assess the spatial learning and memory of APP/PS1 mice, and the investigator was blinded to the groups for the behavioral assessments. Briefly, the Morris water maze apparatus consisted of a pool with a diameter of 120 cm and a height of 40 cm filled with water opaque water colored with milk powder. The water temperature was maintained at 22 ± 1 °C. The pool was surrounded by a white curtain. An escape platform (20 cm in diameter) was submerged 0.5 cm under the water level and located in the center of the target quadrant. Dark posters, different in shape (one per wall), provided distant landmarks. Mouse behavior was recorded using a video camera connected to a video tracking system (RWD Life Science Co., Ltd, Shenzhen, China). The mice (*n* = 10 per group) were subjected to training and probe tests. The training test consisted of 5 consecutive days (4 trials per day, separated by 1-h intervals). For each trial, the mouse was placed in the water facing the wall at different start locations and was required to locate the submerged platform. The time each mouse took to reach the hidden platform was recorded as the escape latency. If the platform was not located within 60 s, the mouse was gently guided to the platform and allowed to stay on the platform for 30 s. The probe test was performed on the sixth day. During the probe trial, the hidden platform was removed, and the mice were allowed to swim for 60 s. The percentage of time spent in the target quadrant was calculated.

### MicroPET

Brain glucose uptake was evaluated using ^18^F-FDG microPET imaging as described in our previous study [20]. After a 6-h fast, the body weight and blood glucose level of the mice (*n* = 10 per group) were measured. The mice received ^18^F-FDG (200 ± 10 μCi) from the tail vein. The mice were anesthetized at 60 min postinjection with 2% isoflurane using a Matrix VIP 3000 calibrated vaporizer (Midmark, OH, USA) and placed on a scanning bed. PET was performed for 10 min followed by a CT scan using a TransPET Discoverist 180 system (Raycan Technology Co., Ltd, Suzhou, China). Body temperature was maintained at 37 °C with a heating pad during anesthesia. PET image reconstruction was performed using the 3-dimensional ordered-subject expectation maximization method with a voxel size of 0.5×0.5×0.5 mm^3^. CT images were reconstructed using the FDK algorithm with a 256×256×256 matrix. Images were displayed with Carimas software (Turku PET Center, Turku, Finland). The mean standardized uptake value was calculated using the following formula: mean pixel value with the decay-corrected region of interest activity (μCi/kg).

### Elisa

The APP/PS1 cells (*n* = 6 per group) and the hippocampus of mice (*n* = 8 per group) were collected and lysed in RIPA buffer containing phosphatase inhibitor and protease inhibitor cocktail. The homogenates were centrifuged at 20,000 rpm for 10 min at 4 °C, and the supernatants were pooled for the analysis of soluble Aβ40 and Aβ42. To extract fibrillar and membrane-bound insoluble Aβ40 and Aβ42, the pellets were homogenized in 70% formic acid and centrifuged at 40,000 rpm for 10 min at 4 °C. The supernatants were neutralized with 1 M Tris-base and analyzed for insoluble Aβ40 and Aβ42.

### Transmission electron analysis

The hippocampi (*n =* 3 per group) were fixed in 4% paraformaldehyde and 1% glutaraldehyde in 0.1 M sodium cacodylate buffer (pH 7.2) overnight at room temperature. Following fixation, the hippocampi were treated with reduced 1% osmium tetroxide, followed by 1% tannic acid in 0.1 M sodium cacodylate buffer for 1 h. The hippocampi were then stained with 2% aqueous solution of uranyl acetate for 30 min, dehydrated in a series of graded ethanol concentrations, and processed for enface embedding in PolyBed (Polysciences). Blocks were sectioned at a 90-nm thickness, poststained with Venable’s lead citrate, and viewed with a transmission electron microscope (JEOL, Tokyo, Japan). Images were obtained by observers who were blinded to the experimental groups.

### Immunofluorescence

Brain tissue (*n* = 3 per group) was fixed in 4% paraformaldehyde, dehydrated, and embedded in paraffin followed by dehydration in graded ethanol solutions and in toluene. Coronal slices (5 μm) were cut on a slicer. Immunofluorescence was performed to detect Aβ plaques. The sections were incubated with 1% BSA containing 0.1% Triton X-100 in PBS at room temperature for 1 h and then coincubated with rabbit polyclonal anti-Aβ antibody at 4 °C overnight. The sections were washed three times in PBS and incubated with goat anti-rabbit IgG H&L (Alexa Fluor® 594) at room temperature for 1 h. Cover slips were mounted in Gel Mount (Vectashield, CA, USA). The nuclei were stained with DAPI. The sections were scanned using a Pannoramic MIDI scanner (3DHISTECH, Budapest, Hungary). The percentage of Aβ plaque area in the hippocampus was quantified.

### Western blot analysis

Western blot analysis was conducted as described previously [21]. The APP/PS1 cells (1.5 × 10^4^ per well) were plated in 6-well plates and cultured for another 24 h. The APP/PS1 cells were incubated with LPD (20 μM) or cotreated with LPD (20 μM) and GW9662 (10 μM) or cotreated with LPD (20 μM) and Mdivi-1 (20 μM) for 24 h. The cells (*n* = 6 per group) or frozen hippocampal tissues from mice (*n* = 6 per group) were homogenized in cold RIPA buffer containing a protease inhibitor cocktail, phosphatase inhibitor cocktail, and phenylmethanesulfonylfluoride (Roche, IN, USA) and then centrifuged at 10,000 rpm at 4 °C for 10 min. The supernatants were collected, and the protein concentrations were determined using BCA kits. Equal amounts of protein were separated by electrophoresis in 10% sodium dodecyl sulfate-polyacrylamide gels and transferred to PVDF membranes (Bio-Rad, CA, USA). The membranes were blocked in 5% nonfat milk powder in Tris-buffered saline containing 0.1% Tween-20 (TBST) for 1 h and then incubated at 4 °C overnight with anti-PPARγ, anti-PINK1, anti-p-Parkin(ser65), anti-LC3B, anti-SQSTM1/p62, and anti-beta tubulin antibodies. After rinsing in TBST, the membranes were incubated with goat anti-rabbit IgG H&L (HRP) or goat anti-mouse IgG H&L (HRP) antibodies at room temperature for 1 h. The protein bands were visualized by a ChemiDoc Touch Imaging System (Bio-Rad, CA, USA) using ECL kits and quantified by Molecular Imager Image Lab software (Bio-Rad, CA, USA). All protein band densities were normalized relative to beta tubulin.

### Hematoxylin and eosin (H&E) staining

Liver samples (*n* = 10) were fixed in 4% paraformaldehyde, embedded in paraffin, and sectioned at 5-μm thickness. Tissue sections were stained with hematoxylin and eosin and then scanned using a Pannoramic MIDI scanner (3DHISTECH, Budapest, Hungary).

### Statistical analysis

Data are presented as the means ± SD and were analyzed using IBM SPSS Statistics version 20 (SPSS Inc., IL, USA). The independent samples *t* test was used to compare data between two groups. Comparisons among three or more groups were conducted using a one-way analysis of variance (ANOVA) followed by Tukey’s post hoc test. The statistical significance of the genotype and treatment effects was assessed using two-way ANOVA followed by Tukey’s post hoc test. *P* < 0.05 was considered statistically significant.

## Results

### Ligustrazine piperazine derivative

**Fig. 1:**
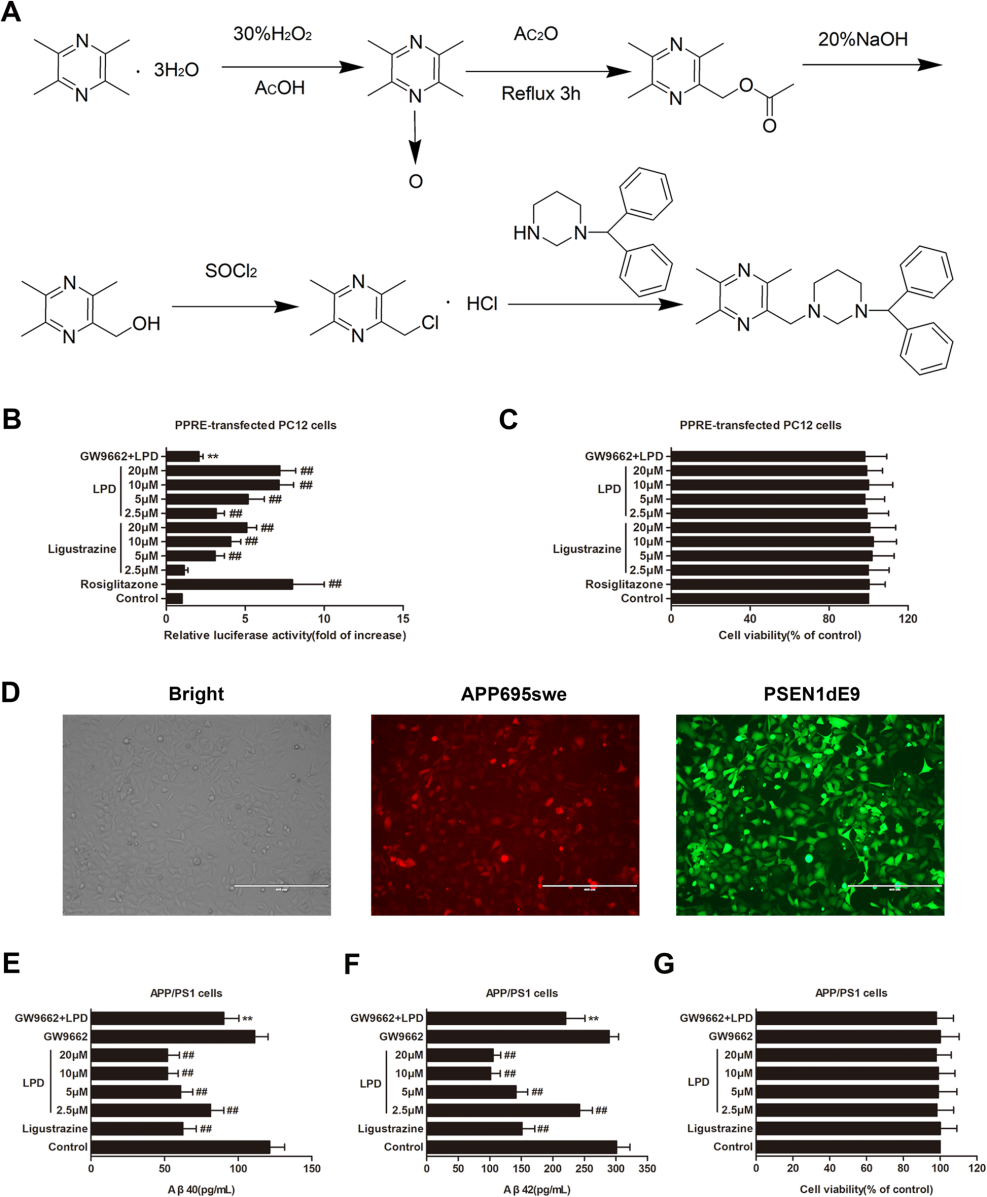
LPD is a novel PPARγ agonist and significantly reduced the levels of Aβ40 and Aβ42 in APP/PS1 cells. **A** Synthesis of ligustrazine piperazine derivative (LPD). **B** Identification of LPD as a novel natural PPARγ agonist using a dual-luciferase reporter assay system. **C** LPD treatment had no significant effect on the viability of PPER-transfected PC12 cells. **D** Fluorescence images of the APP/PS1 stably overexpressing cell line. Scale bar = 400 μm. **E** LPD treatment significantly reduced Aβ40 levels in APP/PS1 cells. **F** LPD treatment substantially reduced Aβ42 levels in APP/PS1 cells. **G** LPD treatment had no significant effect on the viability of APP/PS1 cells. Data are expressed as the means ± SD (*n* = 6 per group) and were analyzed by one-way ANOVA followed by Tukey’s post hoc test. ##, *P* < 0.01 versus control; **, *P* < 0.01 versus LPD treatment

As depicted in Fig. 1A, solvent V ethylacetate to V cyclohexane equals 1:3 for rapid column separation, and a light yellow powder was obtained in 56% yield, m. p. 121–122 °C. LPD was obtained by recrystallization with n-hexane (purity = 98%). IR (KBr), cm^−1^: 2 809.86 (CH); 1 598.06 (C=C); 1 585.41 (C=N). ^1^HNMR (CDCl3), δ: 7.41 (d, 4H, J = 7.75 Hz, Ar-H); 7.26 (t, 4H, J = 7.70 Hz, Ar-H); 7.16 (t, 2H, J = 7.46 Hz, Ar-H); 4.22 (1H, CH); 2.30–3.62 (m, 10H, CH_2_); 2.56 (s, 3H, CH_3_); 2.52 (s, 3H, CH_3_); 2.47 (s, 3H, CH_3_). ESI-MS: 387.5 (M+1).

### LPD is a novel PPARγ agonist

In this study, a dual-luciferase reporter assay system was used to determine whether LPD is a novel PPARγ agonist. As depicted in Fig. 1B, with rosiglitazone as a positive control, both ligustrazine and LPD caused a concentration-dependent increase in PPRE-driven luciferase activity in PC12 cells (*P* < 0.01). The PPRE-driven luciferase activity dramatically increased to sevenfold after LPD (20 μM) treatment for 12 h (*P* < 0.01). Conversely, a statistically significant decrease in PPRE-driven luciferase activity was observed in PPRE-transfected PC12 cells cotreated with LPD and the selective PPARγ antagonist GW9662 (*P* < 0.01). However, the viability of PPRE-transfected PC12 cells was not affected by ligustrazine or LPD or GW9662 (*P* > 0.05, Fig. 1C).

### LPD treatment decreased the levels of Aβ40 and Aβ42 in APP/PS1 cells in a PPARγ-dependent manner

As depicted in Fig. 1D, stable APP/PS1-overexpressing cell lines were established. Treatment with ligustrazine (20 μM) for 24 h significantly reduced the levels of Aβ40 and Aβ42 in APP/PS1 cells. Moreover, incubation of APP/PS1 cells with LPD resulted in a dose-dependent decrease in the levels of Aβ40 and Aβ42 (*P* < 0.01, Fig. 1E, F). In contrast, a significant increase in Aβ40 and Aβ42 levels was observed in APP/PS1 cells cotreated with LPD and GW9662 (*P* < 0.01). However, the viability of stable APP/PS1 overexpressing cell lines was not affected by ligustrazine or LPD or GW9662 (*P* > 0.05, Fig. 1G).

### LPD treatment ameliorated cognitive decline in APP/PS1 mice in a PPARγ-dependent manner

**Fig. 2:**
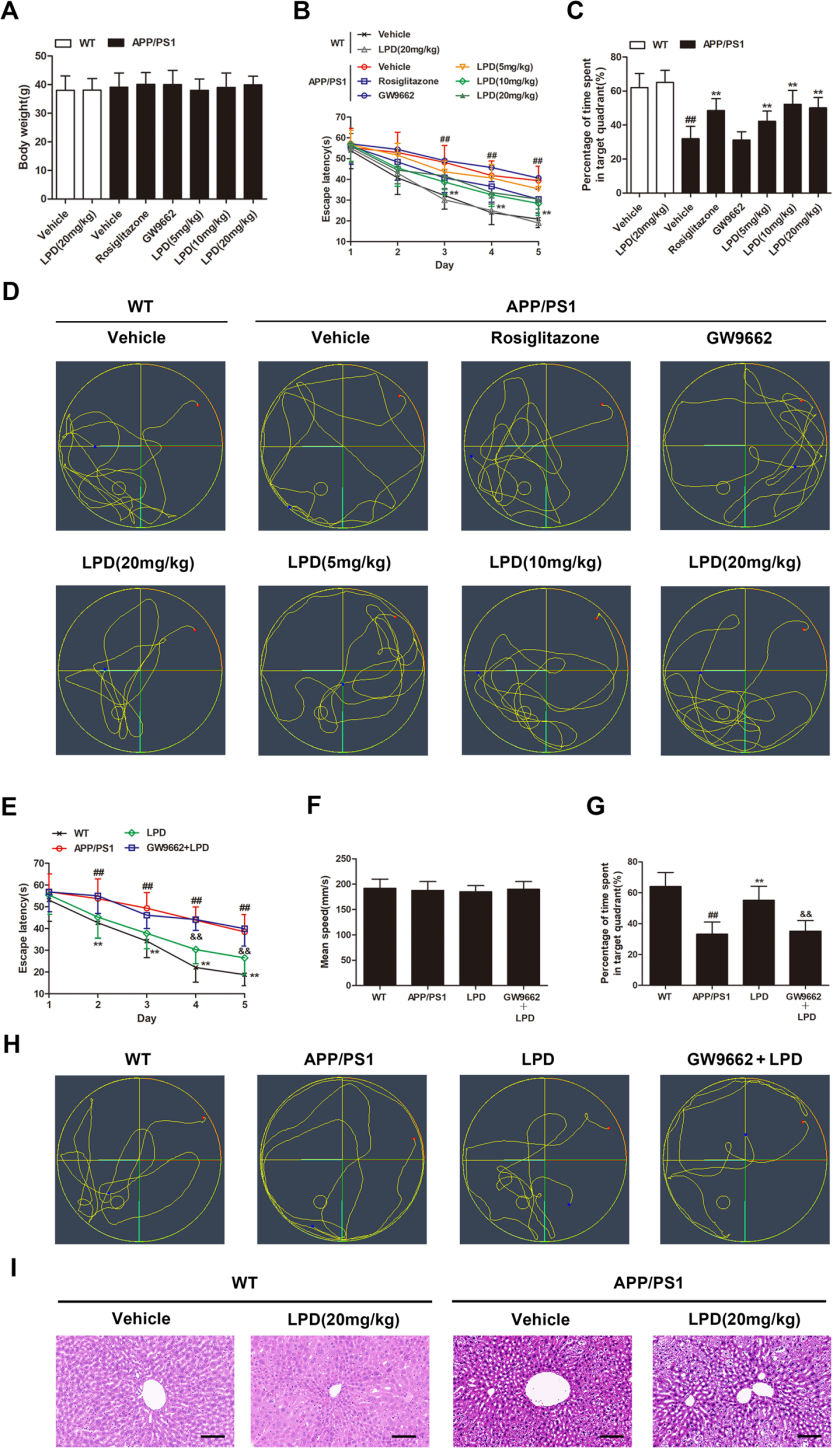
LPD treatment reversed spatial learning and memory. **A** No difference in body weight was observed in mice across treatments and genotypes. **B** LPD treatment dose-dependently decreased the escape latency of APP/PS1 mice in the training trials. **C** LPD treatment markedly increased the target quadrant time of APP/PS1 mice in the probe trials. **D** The swimming path of mice in the probe test. **E** LPD treatment PPARγ-dependently decreased escape latency in the training trials. **F** There was no difference seen in the swimming speed of mice among treatments and genotypes. **G** Cotreatment with GW9662 abolished the LPD-mediated increase in the target quadrant time of APP/PS1 mice in the probe trials. **H** The swimming path of mice in the probe test. **I** There was no significant change in livers of WT and APP/PS1 mice treated with LPD. Scale bar = 100 μm. Data are expressed as the means ± SD (*n* = 10 per group) and were analyzed by two-way ANOVA followed by Tukey’s post hoc test. ##, *P* < 0.01 versus WT; **, *P* < 0.01 versus APP/PS1 mice. &&, *P* < 0.01 versus LPD-treated APP/PS1 mice

As depicted in Fig. 2A, there was no difference seen in the body weight of mice among treatments and genotypes (*P* > 0.05). The effects of LPD on the spatial learning and memory of APP/PS1 mice were evaluated using the Morris water maze. At the age of 9 months, the latency to locate the hidden platform for APP/PS1 mice was significantly longer than that for WT mice (*P* < 0.01, Fig. 2B). However, we found that intragastric administration of LPD for 3 months significantly reduced the escape latency of APP/PS1 mice (*P* < 0.01, Fig. 2B). In the probe test, APP/PS1 mice demonstrated a decreased time in the target platform quadrant compared with WT mice (*P* < 0.01, Fig. 2C, D). Treatment with LPD for 3 months significantly increased the target quadrant time (*P* < 0.01, Fig. 2C, D), using rosiglitazone as a positive control. The protective effect of LPD reached a maximum at a dose of 10 mg/kg. Therefore, treatment with LPD (10 mg/kg) for 3 months was selected for further experiments. Moreover, treatment with GW9662 (5 mg/kg) alone had no significant effect on the escape latency (*P* > 0.05, Fig. 2A) or the target quadrant time of APP/PS1 mice (*P* > 0.05, Fig. 2C, D). In addition, treatment with LPD (20 mg/kg) had no significant effect on the escape latency (*P* > 0.05, Fig. 2A) or the target quadrant time of WT mice (*P* > 0.05, Fig. 2C, D). However, cotreatment with GW9662 and LPD significantly increased the escape latency (*P* < 0.01, Fig. 2E) and decreased the target quadrant time compared with LPD treatment alone (*P* < 0.01, Fig. 2G, H). These results suggested that LPD could attenuate cognitive dysfunction in APP/PS1 mice in a PPARγ-dependent manner. No difference in swimming speed was identified in the probe test among treatments and genotypes (*P* > 0.05, Fig. 2F). To evaluate the effect of LPD treatment on pathomorphology of WT and APP/PS1 mice, the liver sections were stained with H&E. In livers of WT mice, the cells were complete, clear, and regular (Fig. 2I). However, in livers of APP/PS1 mice, the boundary of cells was not clear, and the vacuoles of lipid droplets were observed (Fig. 2I). In addition, there was no significant change in livers of WT and APP/PS1 mice treated with LPD at a dose of 20 mg/kg (Fig. 2I).

### LPD treatment attenuated amyloid pathologies in APP/PS1 mice in a PPARγ-dependent manner

**Fig. 3:**
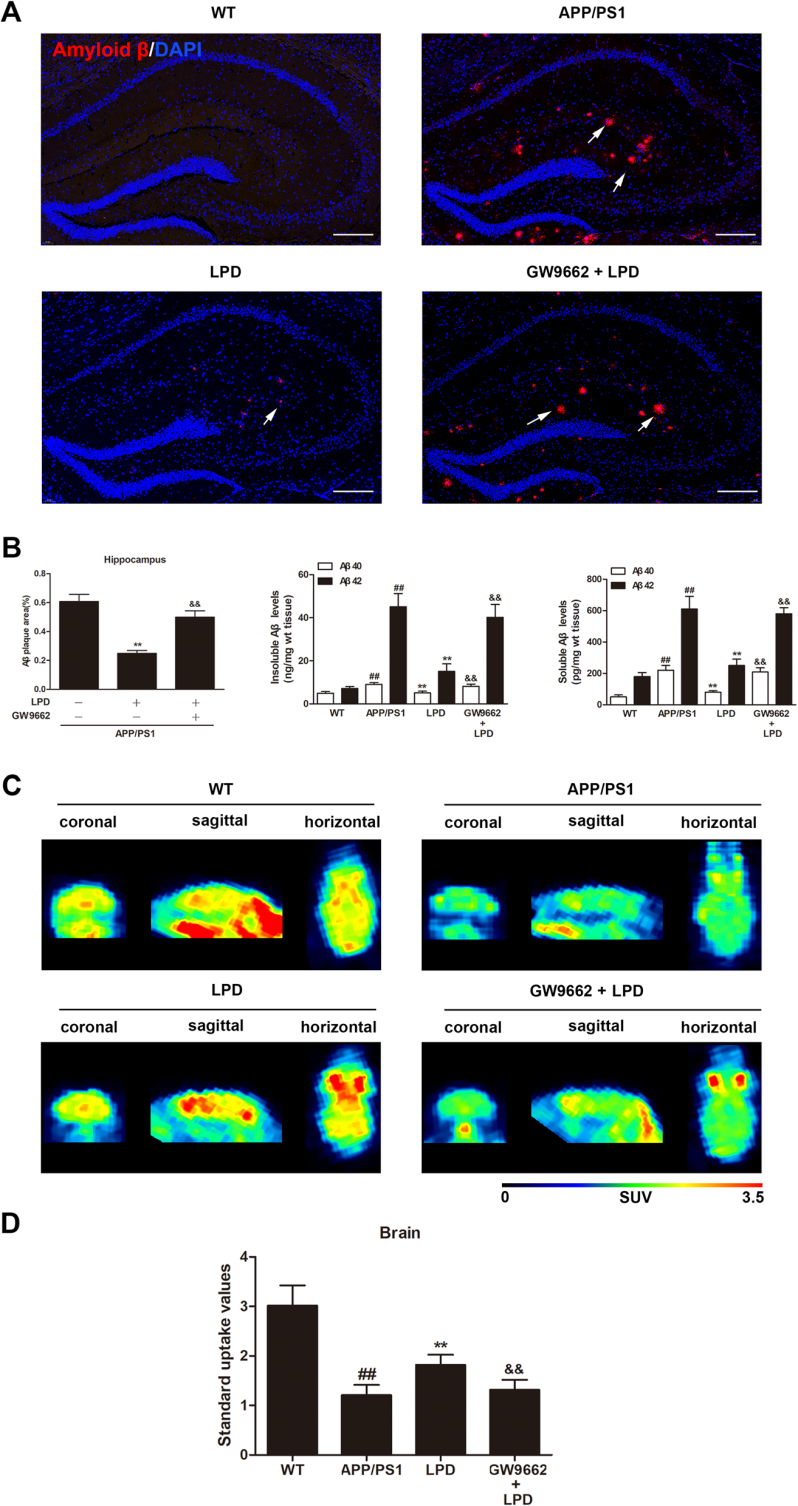
LPD treatment reduced Aβ plaques and increased brain glucose uptake in a PPARγ-dependent manner. The brain sections (*n* = 3 per group) were stained by immunofluorescence using an anti-Aβ antibody. **A** Immunofluorescence images of Aβ plaques in the hippocampus. Scale bar = 200 μm. Arrows indicate Aβ plaques. **B** The percentage of Aβ plaque area in the hippocampus was quantified. The insoluble and soluble forms of Aβ40 and Aβ42 in the hippocampus (*n* = 8 per group) were detected by ELISA. **C** MicroPET imaging with ^18^F-FDG was used to investigate the effect of LPD on brain glucose uptake in APP/PS1 mice (*n* = 10 per group). **D** LPD treatment markedly increased the standardized uptake value in APP/PS1 mice. Data are expressed as the means ± SD and were analyzed by two-way ANOVA followed by Tukey’s post hoc test. ##, *P* < 0.01 versus WT; **, *P* < 0.01 versus APP/PS1 mice. &&, *P* < 0.01 versus LPD-treated APP/PS1 mice

Immunofluorescence was used to assess the effects of LPD on the distribution and morphology of Aβ plaques in brain sections. We observed that the number of Aβ plaques in the hippocampus of APP/PS1 mice was significantly increased compared to that in WT mice (Fig. 3A). In contrast, the area occupied by the Aβ plaques was significantly reduced in the hippocampus from LPD-treated APP/PS1 mice (*P* < 0.01, Fig. 3A, B). As shown in Fig. 3B, APP/PS1 mice exhibited a significant increase in the levels of insoluble and soluble forms of Aβ40 and Aβ42 in the hippocampus compared to levels in WT mice (*P* < 0.01). In contrast, we observed a significant reduction in the levels of insoluble and soluble forms of Aβ40 and Aβ42 in the hippocampus of APP/PS1 mice compared with vehicle-treated mice (*P* < 0.01, Fig. 3B). However, cotreatment with LPD and GW9662 markedly increased the area of Aβ plaques and the levels of insoluble and soluble forms of Aβ40 and Aβ42 in the hippocampus of APP/PS1 mice compared with LPD treatment alone (*P* < 0.01, Fig. 3A, B). These results suggested that LPD could attenuate amyloid pathologies in APP/PS1 mice in a PPARγ-dependent manner.

### LPD treatment increased brain glucose uptake in APP/PS1 mice in a PPARγ-dependent manner

There is some evidence that cerebral glucose hypometabolism is associated with an increased risk of AD [1, 4]. Based on ^18^F-FDG microPET imaging, brain glucose uptake was significantly decreased in APP/PS1 mice compared with WT mice (*P* < 0.01, Fig. 3C, D), and this difference was reversed by LPD treatment (*P* < 0.01, Fig. 3C, D). However, GW9662 cotreatment alleviated the elevated ^18^F-FDG uptake in the brains of LPD-treated APP/PS1 mice (*P* < 0.01, Fig. 3C, D).

### LPD treatment increased hippocampal mitophagy in APP/PS1 mice in a PPARγ-dependent manner

**Fig. 4:**
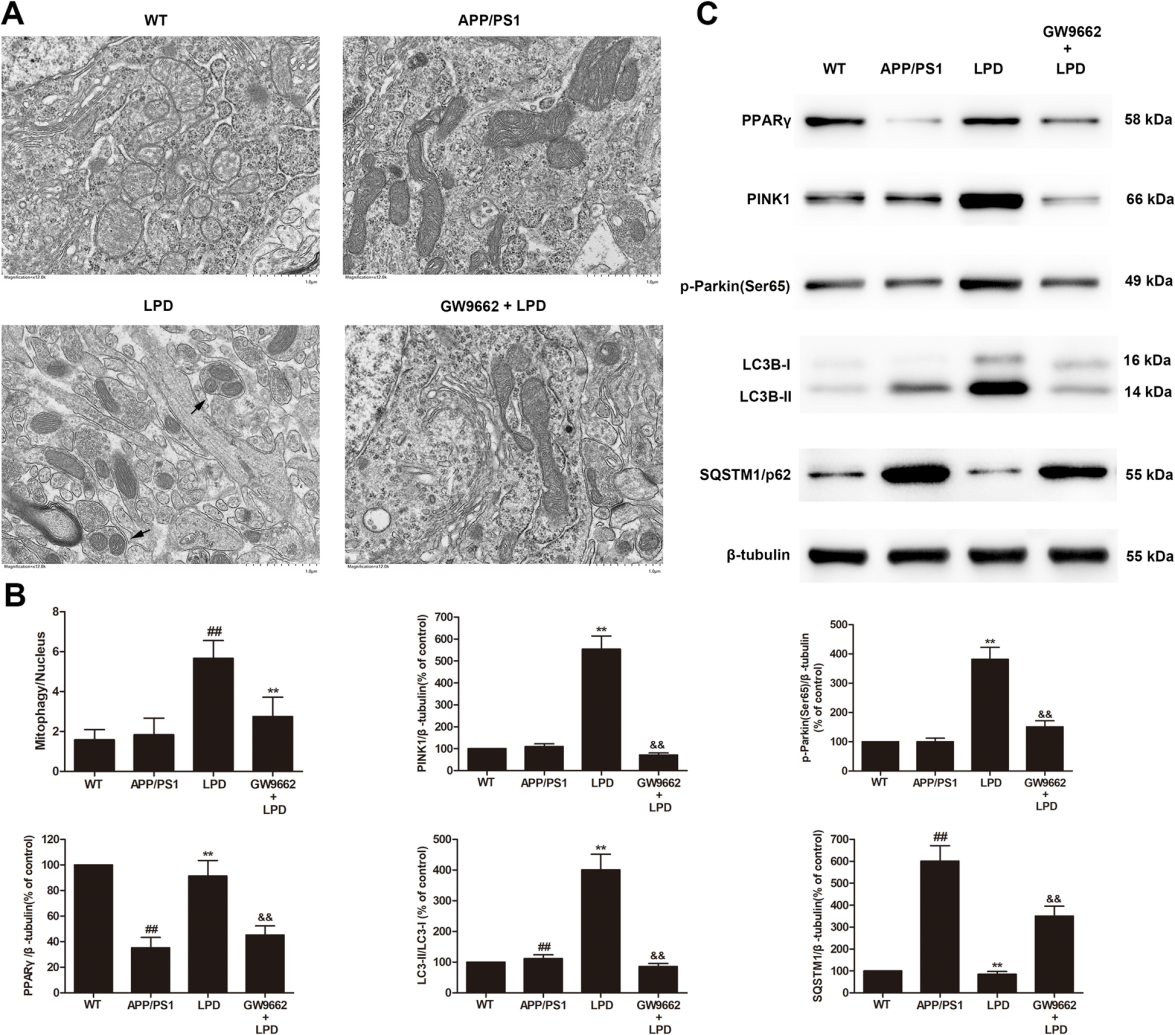
LPD treatment enhanced PINK1/Parkin-mediated mitophagy in vivo in a PPARγ-dependent manner. **A** LPD treatment markedly increased mitophagy in the hippocampi of APP/PS1 mice (*n* = 3 per group). Arrows indicate mitophagy. **B** Quantitative analysis of mitophagy and protein expression. **C** Representative western blot images of PPARγ, PINK1, p-Parkin (Ser65), LC3-II, LC3-I, and SQSTM1/p62 in the hippocampi of mice (*n* = 6 per group). Data are expressed as the means ± SD and were analyzed by two-way ANOVA followed by Tukey’s post hoc test. ##, *P* < 0.01 versus WT; **, *P* < 0.01 versus APP/PS1 mice. &&, *P* < 0.01 versus LPD-treated APP/PS1 mice

Transmission electron microscopy (TEM) was used to examine the effect of LPD on mitophagy in the hippocampi of APP/PS1 mice. Hippocampal neurons from APP/PS1 mice displayed altered mitochondrial morphology characterized by excessive mitochondrial damage in comparison to those from WT mice (Fig. 4A). Induction of mitophagy by LPD resulted in the clearance of damaged mitochondria in APP/PS1 mice (*P* < 0.01, Fig. 4A, B). However, blockade of PPARγ with GW9662 markedly decreased the number of mitophagy events in hippocampal neurons (*P* < 0.01, Fig. 4A, B).

### LPD treatment activated hippocampal PINK1/Parkin signaling in APP/PS1 mice in a PPARγ-dependent manner

To understand the molecular mechanisms responsible for the neuroprotective effects of LPD, PPARγ and potential downstream signaling pathways were investigated. Protein expression of PPARγ was significantly decreased in the hippocampus in APP/PS1 mice compared with WT mice (*P* < 0.01, Fig. 4B, C). However, protein levels of PPARγ, PINK1, and p-Parkin (Ser65) were markedly increased in hippocampal lysates from LPD-treated APP/PS1 mice (*P* < 0.01, Fig. 4B, C). Moreover, LPD treatment significantly increased the LC3-II/LC3-I ratio and decreased SQSTM1/p62 expression (*P* < 0.01, Fig. 4B, C). However, coadministration with GW9662 abolished the effects of LPD on the PPARγ and PINK1/Parkin signaling pathways (*P* < 0.01, Fig. 4B, C).

### LPD treatment induced mitophagy in APP/PS1 cells in a PPARγ-dependent manner

**Fig. 5:**
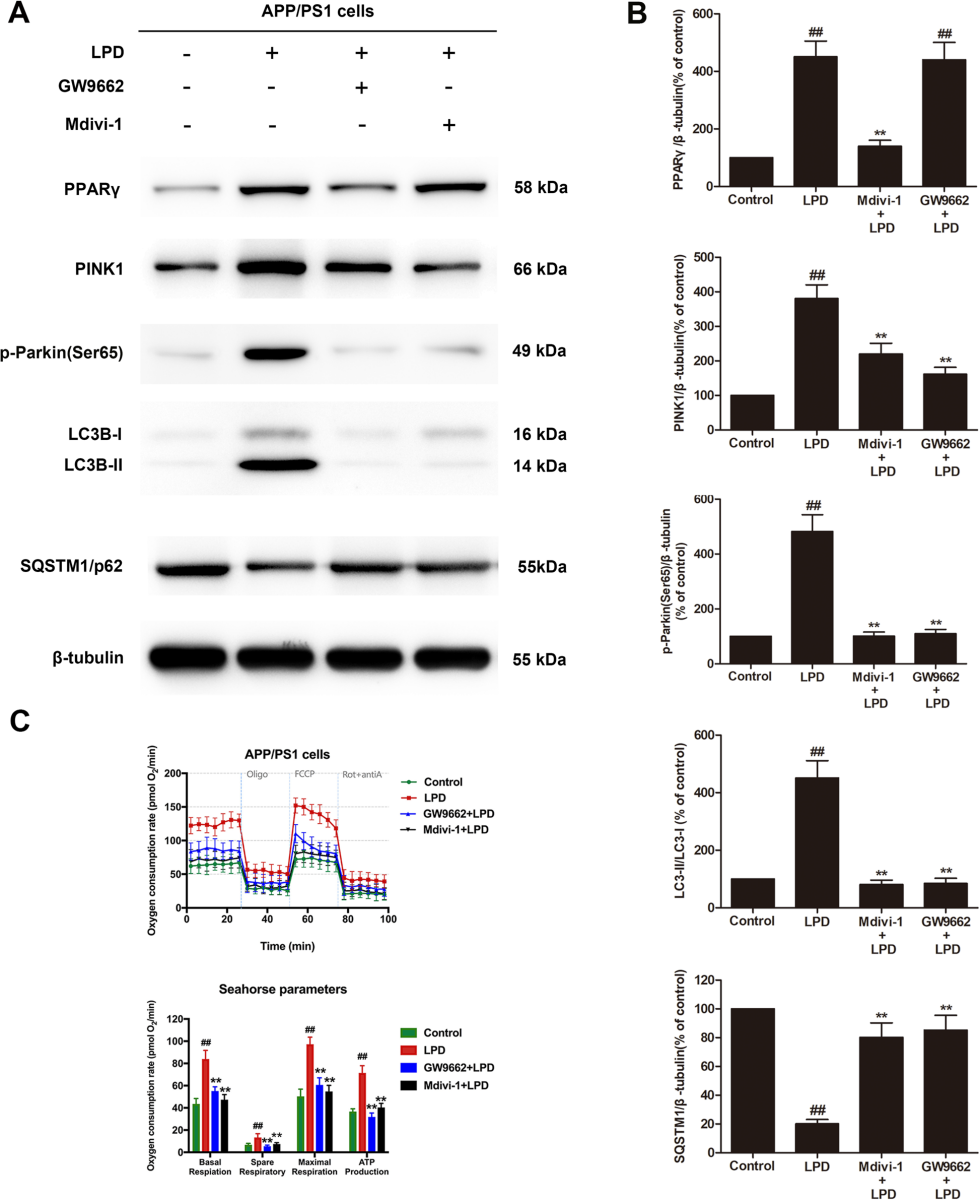
LPD treatment enhanced PINK1/Parkin-mediated mitophagy in vitro in a PPARγ-dependent manner. **A** Cotreatment with GW9662 or Mdivi-1 abolished the effects of LPD on the protein expression of PPARγ, PINK1, p-Parkin (Ser65), LC3-II, LC3-I, and SQSTM1/p62. **B** Quantitative analysis of mitophagy and protein expression. **C** LPD treatment significantly increased OCR, ATP production, maximal respiration, spare respiratory capacity, and basal respiration in APP/PS1 cells. Data are expressed as the means ± SD (*n* = 6 per group) and were analyzed by one-way ANOVA followed by Tukey’s post hoc test. ##, *P* < 0.01 versus control; **, *P* < 0.01 versus LPD treatment

Western blot analysis showed significant increases in the protein expression of PPARγ, PINK1, and p-Parkin (Ser65) in APP/PS1 cells after LPD treatment (*P* < 0.01, Fig. 5A, B). A significant increase in the ratio of LC3-II/LC3-I and a significant decrease in SQSTM1/p62 expression was also observed in APP/PS1 cells treated with LPD (*P* < 0.01, Fig. 5A, B). In contrast, significant decreases in the expression levels of PPARγ, PINK1, and p-Parkin (Ser65) and the ratio of LC3-II/LC3-I, as well as a significant increase in SQSTM1/p62 expression, were observed in APP/PS1 cells cotreated with LPD and GW9662 or the mitophagy inhibitor Mdivi-1 (*P* < 0.01, Fig. 5A, B). Moreover, APP/PS1 cells exposed to LPD had significantly higher OCRs than control cells (*P* < 0.01, Fig. 5C). LPD treatment markedly increased the basal respiration, spare respiratory capacity, maximal respiration, and ATP production in APP/PS1 cells (*P* < 0.01, Fig. 5C). However, cotreatment with LPD and GW9662 or Mdivi-1 abrogated the effects of LPD on mitochondrial function in APP/PS1 cells (*P* < 0.01, Fig. 5C).

## Discussion

Abnormalities in brain glucose metabolism may be intrinsic to AD pathogenesis [4, 15]. PPARγ is of high importance due to its crucial role in glucose metabolism [5]. The ligands for PPARγ, including the thiazolidinedione class of antidiabetic drugs, could reverse cognitive deficits in rodent models of AD [31]. Our previous study confirmed that the endogenous PPARγ agonist 15d-PGJ2 improved cognitive dysfunction in APP/PS1 mice [21]. Interestingly, in the present study, we found that LPD is a novel PPARγ agonist, as evidenced by dual-luciferase reporter assays. We further examined the effect of LPD-induced PPARγ activation on AD pathologies and behavioral phenotypes in APP/PS1 mice. Our data showed that LPD treatment conferred significant improvements in spatial learning and memory in APP/PS1 mice, in a PPARγ-dependent manner, using the Morris water maze test. LPD treatment effectively diminished several markers of AD pathology, including amyloid plaque burden and soluble and insoluble Aβ40 and Aβ42.

Increasing evidence has demonstrated that inefficient glucose utilization leads to synaptic dysfunction, neuronal death, and ultimately cognitive dysfunction [18, 30]. In the present study, APP/PS1 mice demonstrated a decrease in brain glucose uptake compared to WT mice, and this reduction was reversed by the administration of LPD. The accumulation of damaged mitochondria is a hallmark of AD [34]. Mitophagy is a selective form of macroautophagy in which mitochondria are preferentially targeted for degradation at the autophagolysosome [33]. Given their canonical function in mitophagy, the neuroprotective functions of PINK1 and Parkin have largely been attributed to their role in promoting mitochondrial turnover and metabolic homeostasis [36]. To further investigate the potential mechanisms underlying alterations in mitophagy in AD, PINK1/Parkin signaling was investigated. Parkin is an E3 ubiquitin ligase recruited by PINK1 to mitochondria to promote mitophagy in response to chemotherapeutic agents. In this study, LPD treatment increased the expression levels of PINK1 and the phosphorylation of Parkin (Ser65) in vivo and in vitro.

Previous studies have demonstrated that PPARα can activate PINK1/Parkin signaling [25]. Activation of PINK1/Parkin by modulating nuclear receptors, including PPARs, with currently available drugs or new molecules might represent a valid therapeutic target for the treatment of AD [25, 31]. We therefore investigated whether LPD could activate PINK1/Parkin signaling in a PPARγ-dependent manner. In the present study, the PPARγ inhibitor GW9662 abated LPD-mediated activation of PINK1/Parkin signaling. However, LPD-mediated increases in PPARγ expression were not affected by the mitophagy inhibitor Mdivi-1. This may provide important insights into the role of PPARγ in the activation of PINK1/Parkin signaling; however, we are not able to rule out the possibility that LPD can activate PPARα signaling to induce mitophagy.

It is noteworthy that these findings potentially help fill in the gaps in what we know regarding the mechanistic link between PPARγ agonists and the anti-AD effects of natural medicines or traditional Chinese medicines. Our results have important translational implications and set the stage for future studies that may uncover therapeutic interventions targeting brain glucose dysregulation in AD. A limitation of this study is the lack of structure-activity analysis of LPD. The physicochemical properties of LPD are significantly different from ligustrazine and other PPARγ agonists including rosiglitazone and pioglitazone, as well as the antagonist GW9662. LPD is strongly basic due to the presence of the piperazine nitrogens and belongs to the class of lysosomotropic compounds [22–24, 39], suggesting a markedly different intracellular distribution and a possible existence of an alternative molecular mechanism for LPD. The structure-activity analysis of LPD represents an important focus for future studies.

In conclusion, LPD ameliorated cognitive deficits by enhancing brain glucose uptake through activation of PPARγ-dependent mitophagy in APP/PS1 mice.

## Data Availability

Data are available upon reasonable request.
